# Mood Sensitivity to Seasonal Changes in African College Students Living in the Greater Washington D.C. Metropolitan Area

**DOI:** 10.1100/tsw.2007.114

**Published:** 2007-05-01

**Authors:** Alvaro Guzman, Kelly J. Rohan, Samina M. Yousufi, Minh-Chau Nguyen, Michael A. Jackson, Joseph J. Soriano, Teodor T. Postolache

**Affiliations:** ^1^ Mood and Anxiety Program, Department of Psychiatry, University of Maryland, MSTF Building, Room 502, 685 West Baltimore Street, Baltimore, MD 21201, USA; ^2^ Residency Training Program, St. Elizabeths Hospital, 2700 Martin Luther King Avenue, Washington, D.C. 20032, USA; ^3^ Psychology Department, University of Vermont, John Dewey Hall, 2 Colchester Avenue, Burlington, VT 05405-0134, USA

**Keywords:** seasonality, winter depression, seasonal affective disorder, summer depression, ethnic differences, African immigrants

## Abstract

The purpose of this study was to estimate the degree of seasonality and prevalence of winter- and summer-type seasonal affective disorder (SAD) in African immigrant college students in comparison with African American peers. A convenience sample of 246 African immigrants and 599 African Americans studying in Washington, D.C. completed the Seasonal Pattern Assessment Questionnaire (SPAQ), which was used to calculate a global seasonality score (GSS) and to estimate the prevalence of winter- and summer-type SAD. Degree of seasonality was related to a complex interaction between having general awareness of SAD, ethnicity, and gender. A greater percentage of African students reported experiencing a problem with seasonal changes relative to African American students, and had summer SAD, but the groups did not differ on GSS and winter SAD. African students reported more difficulties with seasonal changes than their African American peers, which could represent a manifestation of incomplete acclimatization to a higher latitude and temperate climate. As Africans also had a greater rate of summer SAD, this argues against acclimatization to heat.

## INTRODUCTION

Seasonality is defined as changes in mood, energy, sleep length, appetite, weight, and social activities across the seasons[1,2]. Rosenthal et al.[3] first described the clinical features of fall/winter depression, called winter-type seasonal affective disorder (SAD). Subsyndromal SAD (S-SAD) is a milder form of SAD with similar symptoms, but less severe impairment[1].

Although winter-type SAD is more prevalent, a summer-type SAD involving recurrent major depression in summer with remission in fall or winter has also been identified[4,5]. There are several distinctions between winter- and summer-type SAD. Whereas winter-type SAD is characterized by atypical depressive symptoms, such as anergia, oversleeping, overeating, carbohydrate craving, and weight gain, summer-type SAD has more typical depressive symptoms, such as agitation, insomnia, and weight loss[6]. Light deficiency, especially shortened photoperiods, is the hypothesized etiological mechanism behind winter-type SAD in vulnerable individuals[7], and bright light treatment is the treatment of choice[3,8,9]. In contrast, some evidence suggests that summer-type SAD appears to be triggered by heat in individuals with thermoregulatory abnormalities and improves with temperature manipulations[4]. In addition, our group has also proposed a spring-type SAD that may be triggered by the massive peak of aeroallergens in spring, largely dominated by the tree pollen, in individuals who have been previously sensitized to tree pollen[10].

Epidemiological studies suggest that the degree of seasonality and individual experiences may be determined by other factors, such as geographic location (i.e., latitude)[3,4,11], ethnocultural factors[1,12], genetic factors[12,13,14,15], or their interactions. To attempt to isolate these influences, researchers have examined the effect of acclimatization and migration on seasonality.

To date, several studies have examined the effect of relocating from lower to higher latitudes on seasonality. For example, Suhail and Cochrane reported an increased prevalence of winter-type SAD among Asian (i.e., Pakistani, Indian, or Bangladeshi) women who were nonindigenous to England, relative to Caucasian and Asian women who were born in England[16]. Similarly, Eagles et al. reported an increased trend towards winter-type SAD among newcomers to Aberdeen, Scotland, compared to the native population[17]. In a North Canada study, Williams and Schmidt found that individuals with winter-type SAD were more likely to have been born at more southern latitudes[18]. Low and Feissner found increased prevalence of winter-type SAD in New England college students who had moved from more southern latitudes[19]. Saarijarvi et al. reported a higher prevalence of winter-type SAD among Finns as compared to indigenous Lapps in Lapland[20].

These studies support the notion that, at high latitudes, winter-type SAD prevalence is higher among newcomers relative to the indigenous population. In addition to migration, another important consideration is length of residency. Although the above studies did not explicitly examine length of residency, the pattern of findings suggests that length of residency may be inversely related to winter-SAD prevalence. However, it remains unknown whether the nonindigenous samples in these studies developed more or less winter-type seasonality with increasing years of residency. The one study that did examine length of residency in the nonindigenous population found an increased prevalence of winter-type SAD in longer-staying (>10 years) as compared to shorter-staying (<2 years) Japanese residents in Sweden[21].

Another important area for further research is seasonality in ethnic groups other than Caucasians. Because non-Caucasian samples have been understudied, it remains unknown whether findings from Caucasian samples generalize to other ethnic groups. One preliminary study in the U.S.[2] found similar rates of winter-type SAD among African American students as those previously reported for Caucasians in the same geographic region[1]. In contrast to studies on Caucasians, Asian studies with Japanese civil servants[22], Filipino city workers[23], Thai residents[24], and Chinese students[25,26] have generally found a higher prevalence of summer-type than winter-type SAD. Because the Thai and Filipino studies were performed at lower latitudes (i.e., warmer climates), the results may be attributed to increased heat.

In light of the above considerations, we now estimated seasonality in African students in comparison with African American students living in the Washington, D.C. metropolitan area. First, we explore the interactive effects of ethnicity and gender on degree of seasonality. In favor of the majority of evidence supporting that individuals who are not indigenous to a high latitude region have higher rates of winter-type seasonality relative to the native population, we predicted that indigenous Africans would report greater seasonality. Consistent with prior work, we expected greater seasonality among females than males[1,12,27,28,29,30,31,32] and among individuals who have heard about SAD (“awareness” of SAD)[1,2].

In addition, we extended our examination of seasonality to include the effect of the perception of seasonal changes as a problem. Thus, first we intended to document the prevalence of winter- and summer-pattern of SAD by ethnicity, gender, and awareness of SAD.

Primarily, we predicted that Africans would more frequently report experiencing seasonal changes as a problem, and have a greater prevalence of winter-type SAD, total winter-type SAD (combined prevalence of winter-type SAD and S-SAD), and winter mood pattern than African Americans.

## METHODS

### Participants

This study was conducted at four undergraduate and graduate academic programs in Washington, D.C. that have a high percentage of African and African American students. African students came from Senegal, Gambia, Sierra Leone, Liberia, Ivory Coast, Ghana, Togo, Benin, Nigeria, Angola, Gabon, and Cameroon. To be included in the study, a potential participant must have identified himself or herself as an indigenous African. For the controls, participants had to identify himself or herself as an African American and must have lived in the study area (D.C. latitude = 40°N ± 5°) for at least 3 years.

### Measures

Since its development, the Seasonal Pattern Assessment Questionnaire (SPAQ)[33] has been used with college students[2,19,24,25,34] with a high degree of test-retest reliability[34]. The SPAQ is a self-report instrument, but it generally overestimates SAD when compared to clinical diagnostic interviews[35].

The SPAQ contains questions about changes in mood and behavior across the seasons. Six items (i.e., mood, sleep, appetite, weight, energy, social activities) are rated on a 0 (no change) to 4 (extreme change) scale, and are summed to compute a global seasonality score (GSS)[1]. An affirmative response to the question "If you experience changes with the seasons, do you feel that these are a problem for you?" is used to identify respondents who perceive seasonality as a problem.

In accordance with Magnusson and Stefansson[12], we used the following SPAQ-derived criteria for a SAD diagnosis: (1) GSS $\geq 11$, (2) reporting that seasonal changes pose a problem, and (3) rating the problem severity as at least moderate. S-SAD criteria on the SPAQ are: (1) GSS = 9 or 10, with a problem rating of at least mild; or (2) GSS > 11, with a problem rating of not more than mild. In response to the SPAQ question "At what time of year do you feel worst?", a response of December, January, and/or February adds the winter-type specifier to a SAD or S-SAD diagnosis. Feeling worst during June, July and/or August warrants the summer-type specifier.

### Procedure

The protocol was approved by the Institutional Review Board of the Department of Mental Health of the District of Columbia. Students signed an informed consent document after a full explanation of the study. The SPAQ was administered to students in a classroom setting.

### Statistical Analyses

To examine the impact of ethnicity, gender, and general awareness of SAD on seasonality, we performed an analysis of variance (ANOVA) with GSS as the dependent variable and ethnicity, gender, and awareness as independent factors. Because these relations have not been tested in prior studies, we used two-tailed tests in all analyses. To evaluate the frequency of perceiving seasonal changes as a problem in

African students in comparison with African American students, we performed a Pearson Chi-Square analysis. We descriptively compared rates of winter- and summer-type SAD, total SAD, and mood pattern in African vs. African American students. Chi-square analyses were conducted to test the hypothesized pattern of ethnic differences in winter- and summer-type diagnoses.

## RESULTS

### Demographic Characteristics

The resulting student sample contained 246 Africans and 599 African Americans. As our sample contains many nontraditional students, the average age of Africans was 30.5 years (SD = 10) with a range of 18–55 years, whereas the average age of African Americans was 29.5 years (SD = 10) with a range of 16–67 years. The average duration of residence in the study area was 7.5 years (SD = 5.8) for African and 23.3 years (SD = 11.7) for African American students. Regarding awareness of SAD, 26.8% of Africans and 20.4% of African Americans reported having heard about SAD.

### Seasonality and Ethnicity, Gender, and Awareness of SAD

Table 1 presents GSS by ethnicity, gender, and awareness of SAD. The ethnicity × gender × awareness of SAD interaction was significant for GSS, $F\ (1, 750) = 4.30$, $p = 0.03$. To extricate the three-way interaction, we performed paired post-hoc comparisons using a Bonferroni correction for multiple comparisons with $p$ set at 0.05. African males who reported an awareness of SAD obtained significantly higher seasonality scores than African females who reported an awareness of SAD, $p<0.05$. African females with awareness of SAD, $p<0.05$, evidenced lesser seasonality relative to African American females with awareness of SAD. Among males, Africans who were aware of SAD reported more seasonality than African Americans who were aware of SAD, $p<0.05$. No other significant differences between the cells were found. In contrast to previous studies in the general population, GSS did not correlate negatively with age in our sample, $r = 0.011$, ns.

**TABLE 1**
**GSS by Ethnicity and Gender**

| | Africans | | | African Americans | | |
|---|---|---|---|---|---|---|
| | **M** | **SEM** | **No.** | **M** | **SEM** | **No.** |
| Females | | | | | | |
| Aware of SAD | 7.629 | 0.941 | 31 | 10.385 | 0.626 | 70 |
| Unaware of SAD | 8.286 | 0.605 | 75 | 8.140 | 0.308 | 289 |
| Males | | | | | | |
| Aware of SAD | 10.523 | 1.144 | 21 | 8.681 | 0.790 | 44 |
| Unaware of SAD | 8.066 | 0.636 | 68 | 7.601 | 0.424 | 153 |

### Problem with Seasonal Changes by Ethnicity

A significantly greater percentage (31.9%) of African students reported experiencing a problem with seasonal changes relative to African American students (19.4%), $X^2\ (1, N = 796) = 14.443$, $p < 0.001$.

## Prevalence of SAD and Seasonality by Ethnicity and Gender

Prevalence estimates for winter- and summer-type SAD, total SAD, and mood pattern by ethnicity and gender are presented in Table 2. African and African American students did not differ on winter-type SAD, $X^2$ (2, N = 753) = 0.140, ns; winter-type total SAD, $X^2$ (1, N = 753) = 0.083, ns; or winter mood pattern prevalence, $X^2$ (2, N = 786) = 0.775, ns. Using a median split for years of residency in the study area, short- (<5 years) and long-staying (≥5 years) Africans did not differ in prevalence of winter-type SAD, $X^2$ (2, N = 167) = 3.925, ns, or in winter-type total SAD, $X^2$ (2, N = 167) = 1.619, ns. African students had significantly greater prevalence of summer-type SAD relative to African American students, Fisher's exact test, $p = 0.045$, but the ethnic groups did not differ on total summer-type SAD, $X^2$ (N =551) = 0.842, ns, or summer mood pattern, $X^2$ (N =786) = 1.201, ns.

**TABLE 2**
**Prevalence Estimates for Winter- and Summer-Type SAD, Total SAD, and Mood Pattern**

| | **Africans (*N* = 243)** | **African Americans (*N* = 539)** |
|---|---|---|
| Winter-type SAD, No. (%) | 13/214 (6.08%) | 29/539 (5.38%) |
| Females, No. (%) | 10/119 (8.40%) | 23/349 (6.59%) |
| Males, No. (%) | 2/93 (2.12%) | 6/180 (3.33%) |
| Summer-type SAD, No. (%) | 5/213 (2.35%) | 3/538 (0.55%) |
| Females, No. (%) | 2/120 (1.66%) | 1/349 (0.28%) |
| Males, No. (%) | 3/91 (3.29%) | 2/180 (1.11%) |
| Total SAD, winter, No. (%) | 30/214 (14.01%) | 80/539 (14.84%) |
| Females, No. (%) | 17/119 (14.28 %) | 56/350 (16.0%) |
| Males, No. (%) | 12/93 (12.90%) | 21/181 (11.60%) |
| Total SAD, summer, No. (%) | 12/213 (5.63%) | 22/538 (4.08%) |
| Females, No. (%) | 7/120 (5.83%) | 16/349 (4.58%) |
| Males, No. (%) | 5/91 (5.49%) | 6/181 (3.31%) |
| Winter mood pattern, No. (%) | 78/227 (34.36%) | 174/559 (31.12%) |
| Females, No. (%) | 47/128 (36.71%) | 106/359 (29.53%) |
| Males, No. (%) | 31/97 (31.95%) | 66/194 (34.02%) |
| Summer mood pattern, No. (%) | 11/227 (4.84%) | 18/559 (3.22%) |
| Females, No. (%) | 9/128 (7.03%) | 14/359 (3.90%) |
| Males, No. (%) | 2/97 (2.06%) | 4/194 (2.06%) |

*Note:* Total SAD includes participants who met criteria for either SAD or subsyndromal SAD (S-SAD).

## DISCUSSION

To our knowledge, this is the first study to explore seasonal variations of mood and behavior in African students and compare it with African American students at the same latitude. It is also the largest seasonality study to date on Africans in the U.S. Although they reported more problems with changes in seasons, African students did not differ from African American students on winter-type SAD or total SAD. This is inconsistent with the prior studies supporting that individuals who migrate to a higher latitude region generally demonstrate increased winter-type seasonality relative to the native population[16,17,18,19,20]. It is also possible that immigration stress could worsen the perception of difficulties in general, including those associated with seasonality[36].

Regarding summer seasonality, unexpectedly, African students had a higher rate of summer-type SAD than African Americans. We also revealed a complex pattern of differences when degree of seasonality was examined depending on ethnicity, gender, and having general familiarity with the condition of SAD. Among African American students who reported general awareness of SAD, females obtained higher GSS than males. However, among African students with general awareness of SAD, males reported higher seasonality than females. This reverse pattern of gender differences according to ethnicity with awareness of SAD was unexpected and hypothesis generating for future research.

As a limitation of the present study, our data are entirely based on the retrospective, self-report SPAQ[33]. The SPAQ does not diagnose SAD per se, but gives an estimate of the prevalence and pattern of SAD and subsyndromal SAD symptoms. The SPAQ typically overestimates SAD prevalence when compared to more objective clinical interviews[37]. These biases could be controlled by more prospective, longitudinal design using clinical interviews. As a second limitation, we used a convenience sample of students, rather than a random sample survey of the student population. Our sample may not be representative of the African and African American student population in the D.C. area. Students with high seasonality and/or greater awareness of seasonality and SAD may have been more likely to volunteer. Third, there is a marked genetic[38,39] and cultural heterogeneity within Africans, and between Africans and African Americans, which may impact the biology, experience, appraisal, and reporting of seasonality. Future studies are necessary to determine whether these results generalize to clinical samples and to explore mechanisms underlying these group differences. In the meantime, we need targeted educational efforts in college students to increase awareness of SAD as a treatable condition, which adversely affects mood, well-being and academic performance

## ACKNOWLEDGMENTS

This study was supported by the Department of Mental Health of the District of Columbia (T.T. Postolache, Principal Investigator).
